# Anomalous Left Circumflex Coronary Artery Arising from the Right Pulmonary Artery: A Rare Cause of Aborted Sudden Cardiac Death

**DOI:** 10.7759/cureus.499

**Published:** 2016-02-19

**Authors:** Bo Liu, Dzmitry Fursevich, Matthew C O'Dell, Miguel Flores, Nicholas Feranec

**Affiliations:** 1 Diagnostic Radiology, Florida Hospital-Orlando; 2 College of Medicine, University of Central Florida

**Keywords:** coronary artery anomaly, anomalous coronary artery, vascular malformation, alcapa, bland-garland-white syndrome

## Abstract

We report a case of anomalous origin of the left circumflex coronary artery arising from the right pulmonary artery resulting in stress-induced cardiac arrest. The patient collapsed after running a 5K race and was resuscitated. Subsequent workup revealed the culprit anatomy, which was successfully treated with surgical ligation. To the authors’ knowledge, this is only the second case of this variant coronary anomaly resulting in aborted sudden cardiac death, subsequent surgical ligation, and recovery in a healthy young adult and is the first case treated by ligation alone without coronary bypass.

## Introduction

Anomalies of the coronary arteries are rare, often cited as affecting 1-2% of the population [1-2]. The actual prevalence rate is likely higher, given that many hemodynamically insignificant cases never come to medical attention. Of the hemodynamically significant cases, origin from the pulmonary artery is often diagnosed as a result of severe clinical manifestations, such as myocardial ischemia or left ventricular overload due to shunting shortly after birth. Bland-Garland-White syndrome or anomalous origin of the left coronary artery from the pulmonary artery (ALCAPA) is a well-recognized entity that is thought to constitute 0.25% - 0.5% of congenital heart disease [2]. We describe an even rarer variant of origin of the left circumflex (LCX) coronary artery from the right pulmonary artery (RPA) resulting in sudden cardiac arrest under stress.

## Case presentation

This case report was approved by our institutional review board with a waiver of the requirement for informed consent for publication and was compliant with HIPAA. A healthy 30-year-old female with no significant previous medical history was admitted after a witnessed cardiac arrest. She was running a 5K race when shortly after crossing the finish line, she collapsed into what was described as a “tonic-clonic seizure”. The medical director of the race was on site and found the patient pulseless. An automated external defibrillator was obtained, and the patient was found to be in a “shockable rhythm”. Unfortunately, the electrocardiogram was not available for review. A single shock was delivered resulting in the spontaneous return of circulation. No additional cardiopulmonary resuscitation was needed, and she was transferred to our emergency department for workup and management. Informed patient consent was obtained for treatment.

**Figure 1:**
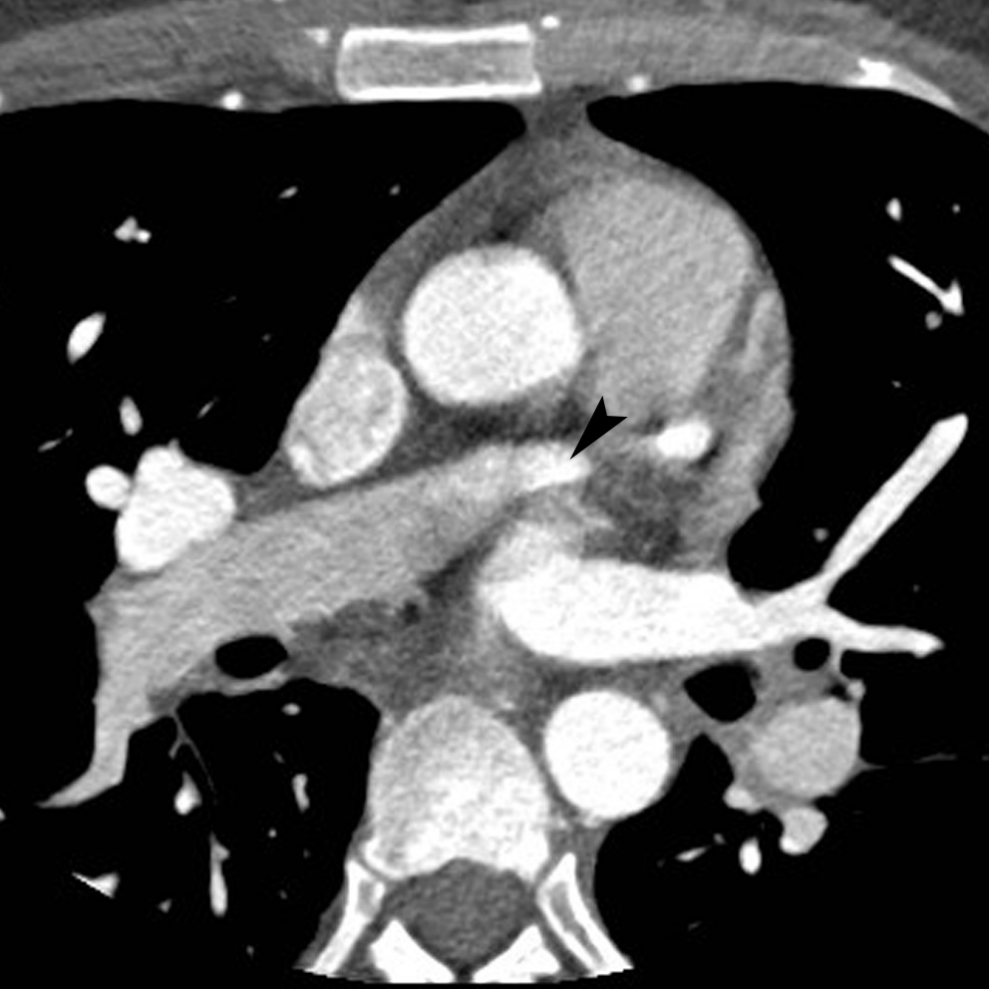
Anomalous LCX artery from RPA Axial coronary CT angiogram image demonstrating the anomalous origin of the left circumflex artery (arrowhead) from the right pulmonary artery. Note the retrograde opacification of the right pulmonary artery.

**Figure 2:**
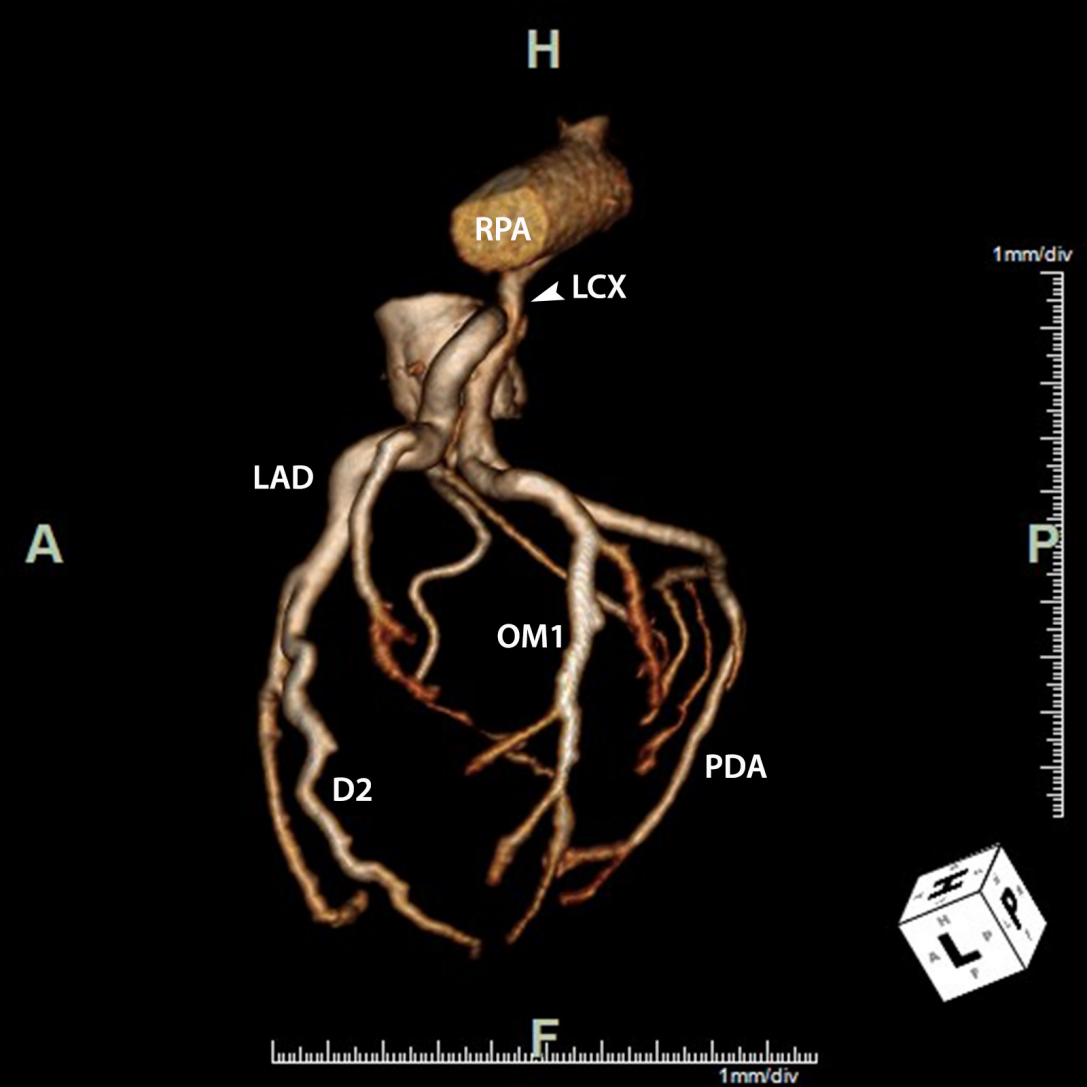
Anomalous LCX artery from RPA Lateral 3D volume-rendered image of the coronary tree demonstrating the anomalous connection of the LCX artery to the right pulmonary artery (RPA) coursing posterior to the proximal left anterior descending (LAD) coronary artery. The LAD coronary artery, second diagonal coronary artery (D2), first obtuse marginal coronary artery (OM1), and posterior descending coronary artery (PDA) are dilated.

**Figure 3:**
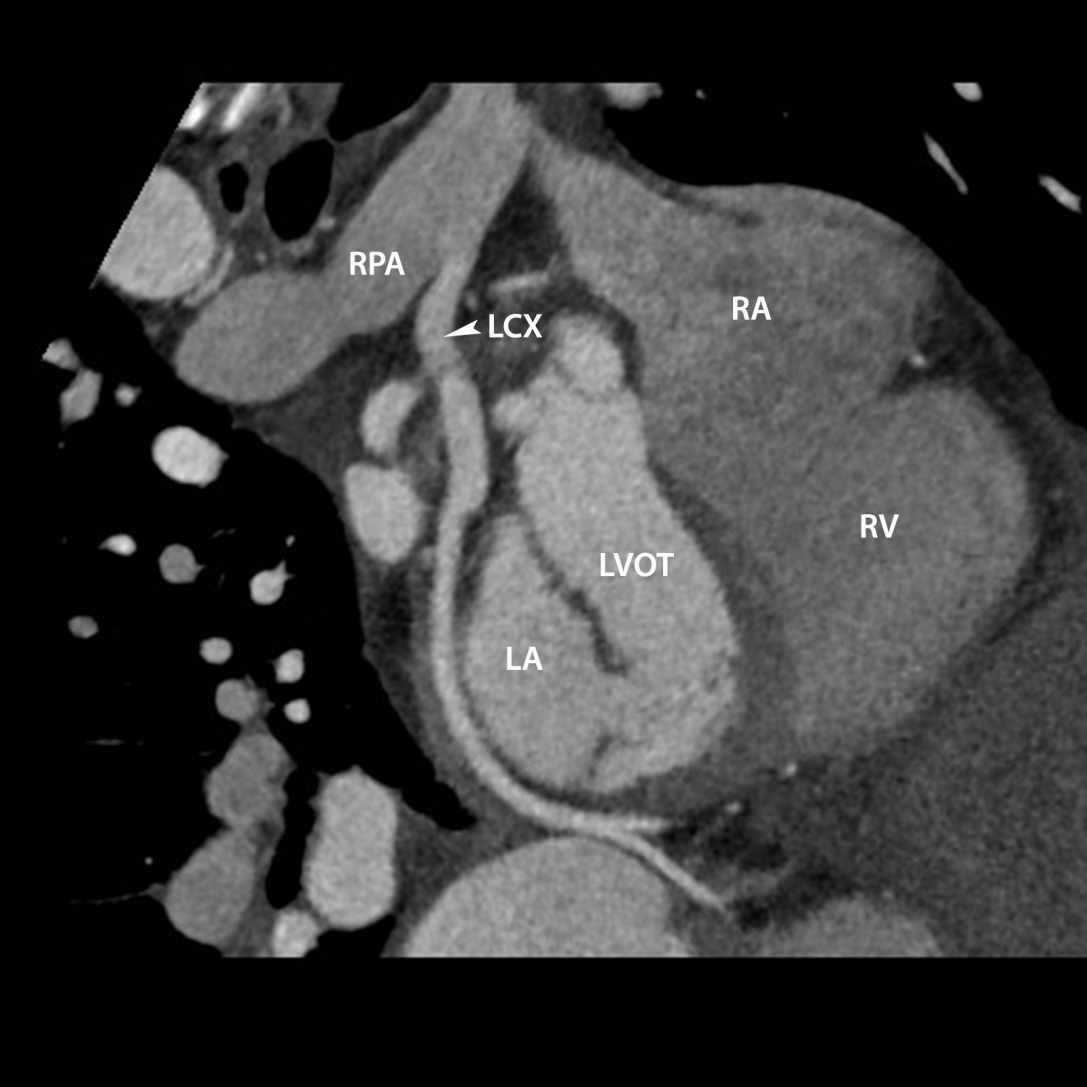
Anomalous LCX artery from RPA Curvilinear reformat of the LCX artery demonstrating its anomalous origin from the RPA. The left atrium (LA), left ventricular outflow tract (LVOT), right atrium (RA), and right ventricle (RV) are also labeled.

Initial workup was significant for elevated white blood cell count of 14,600 cells/mcL, decreased potassium of 3.1 mEq/L, decreased magnesium of 1.7 mg/dL, mildly elevated aspartate transaminase of 92 U/L, mildly elevated alanine transaminase of 80 U/L, and a slightly prolonged QTc of 512 msec. Otherwise, her labs were unremarkable, including negative cardiac enzymes. Imaging workup revealed a normal chest x-ray, normal computed tomography (CT) of the head and cervical spine, normal echocardiogram, and a computed tomography angiogram (CTA) of the chest that was negative for pulmonary embolism. A coronary CTA was obtained to evaluate for congenital coronary anomalies leading to the identification of an anomalous left circumflex coronary artery originating from the proximal right pulmonary artery associated with a dilation of the non-anomalous coronary vessels (Figures 1-3, Video 1).

**Video 1:** Anomalous LCX artery from RPA An axial coronary CT angiogram image demonstrating the anomalous origin of the left circumflex artery from the right pulmonary artery.

**Figure 4:**
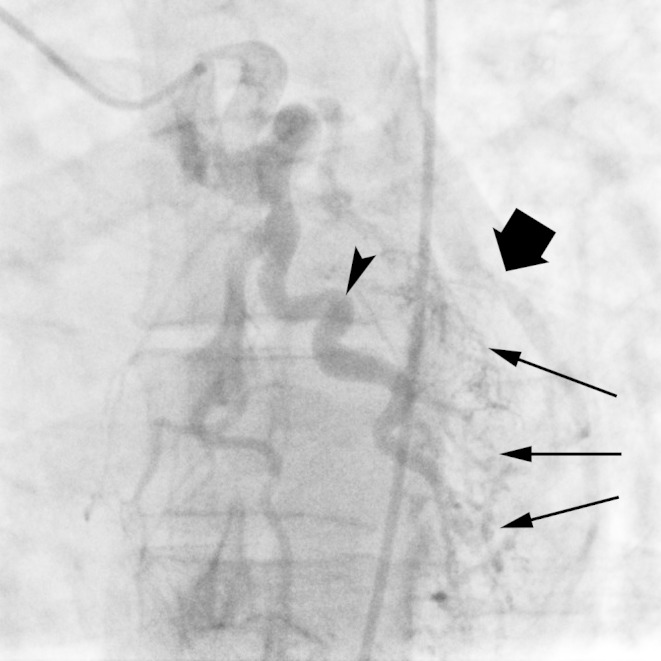
Anomalous LCX from RPA Early diastolic angiographic image demonstrates dilated native LAD (arrowhead) and a network of coronary-coronary collaterals (thin arrows). There is faint retrograde opacification of the the LCX (thick arrow).

**Figure 5:**
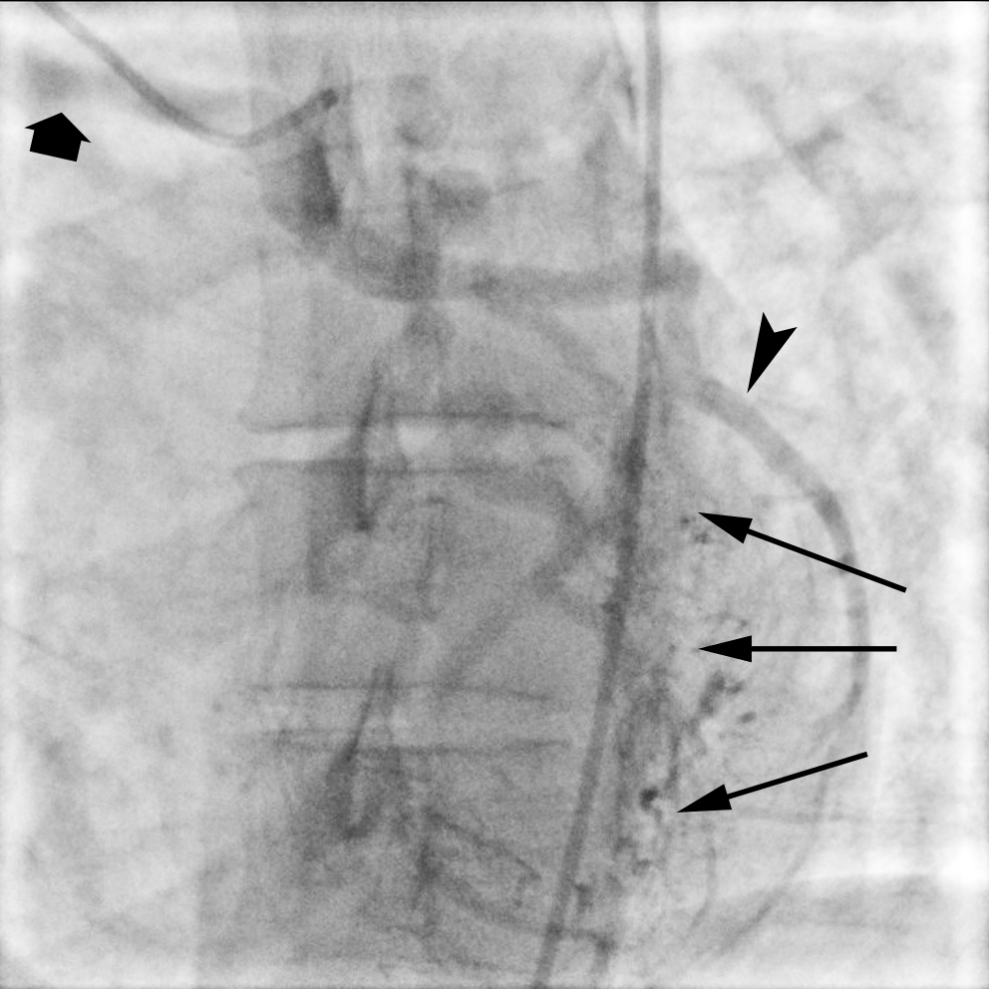
Anomalous LCX artery from RPA A late diastolic angiographic image demonstrates retrograde filling of the LCX artery (arrowhead) from a network of coronary-coronary collateral vessels (thin arrows). Contrast is seen spilling into the right pulmonary artery (thick arrow).

This finding was confirmed on a subsequent catheter coronary angiogram. The angiogram also showed retrograde filling of the anomalous left circumflex coronary artery via a network of collateral vessels from the left anterior descending (LAD) coronary artery and the right coronary artery (RCA) (Figures 4-5).

The patient subsequently underwent open surgical ligation and division of the anomalous left circumflex artery. She experienced no postoperative complications and was discharged eight days later after a comprehensive electrophysiology (EP) study failed to induce any ventricular arrhythmias.

## Discussion

The normal coronary anatomy is characterized by origins of the left main coronary artery (LMCA) and the right coronary artery (RCA) in the left and right sinuses of Valsalva, respectively. The LMCA subdivides into the left anterior descending (LAD) coronary artery and the left circumflex (LCX) coronary artery. These arteries further divide into progressively smaller branches that then progress inward to penetrate the epicardium and supply blood to the transmural myocardium. 

A wide array of coronary artery variants as well as many classification schemes has been described. We prefer the relatively simple classification system proposed by Shriki, et al. dividing coronary artery variants into a) hemodynamically significant anomalies, which may be associated with shunting, ischemia, or sudden cardiac death, and (b) anomalies that are usually not hemodynamically significant [2]. Hemodynamically significant anomalies can be further divided into atresia, origin from the pulmonary artery, interarterial course, and congenital fistula. Of these, Bland-Garland-White syndrome or anomalous origin of the left coronary artery from the pulmonary artery (ALCAPA) is a well-recognized syndrome. Less commonly, the RCA, LAD coronary artery, or LCX coronary artery have been reported to arise from the pulmonary artery in rarer variants of this syndrome.

ALCAPA is usually diagnosed in the neonatal period. As pulmonary vascular resistance falls, there is a progressive retrograde filling of the LMCA via collaterals from the RCA circulation. This results in a steal of blood flow from the myocardium, leading to myocardial ischemia. The left-to-right shunt also leads to left ventricular overload and dilation of the coronary arteries as a result of the increased flow [2]. We believe that this is also the pathophysiology underlying our patient’s sudden cardiac arrest. As to why our patient remained asymptomatic throughout the first 30 years of life, we hypothesize that this is a result of the combination of the degree of coronary-coronary collateralization, the relatively small area of myocardium supplied by the LCX artery, and the lack of significant previous cardiac challenges until the 5K race. We believe the patient's minor electrolyte abnormalities and prolonged QTc on admission did not contribute significantly to the presentation.

Anomalous origin of the LCX artery from the pulmonary artery can be considered an exceedingly rare variant of ALCAPA, with the first adult case reported in 1992 [3]. Since then, only seven additional adult cases have been described with the most common presenting symptom being exertional angina [4-10]. In the reports that included treatment and follow-up data, surgical ligation with coronary bypass was the most commonly performed procedure. Only one of the reported cases presented with a sudden cardiac arrest that was resuscitated and subsequently treated surgically with coronary bypass grafting [10]. The cardiothoracic surgery team was unavailable for comments, but we hypothesize that no bypass was performed in this case, given the rich network of coronary-coronary collaterals.

## Conclusions

In summary, coronary artery anomalies are rare and should be suspected in young adults presenting with cardiac symptoms. The role of the radiologist, cardiologist and electrophysiologist lie in discriminating between the hemodynamically significant and hemodynamically insignificant variants. We hereby describe a case of the LCX artery arising from the RPA, resulting in a stress-induced cardiac arrest. This was successfully treated surgically by ligation and division of the culprit vessel. To our knowledge, this is only the second case of this variant coronary anomaly resulting in aborted sudden cardiac death and the first case repaired with ligation and division alone without coronary bypass grafting.
